# Overexpression of *Prunus mume* Dehydrin Genes in Tobacco Enhances Tolerance to Cold and Drought

**DOI:** 10.3389/fpls.2017.00151

**Published:** 2017-02-07

**Authors:** Fei Bao, Dongliang Du, Yang An, Weiru Yang, Jia Wang, Tangren Cheng, Qixiang Zhang

**Affiliations:** Beijing Key Laboratory of Ornamental Plants Germplasm Innovation and Molecular Breeding, National Engineering Research Center for Floriculture, Beijing Laboratory of Urban and Rural Ecological Environment, Key Laboratory of Genetics and Breeding in Forest Trees and Ornamental Plants of Ministry of Education, School of Landscape Architecture, Beijing Forestry UniversityBeijing, China

**Keywords:** *Prunus mume*, dehydrins, LEA proteins, cold stress, drought tolerance

## Abstract

Dehydrins, known as group 2 or D-11 family late-embryogenesis-abundant (LEA) proteins, play important roles in plant growth and stress tolerance. Six dehydrin genes were previously identified from the genome of *Prunus mume*. In this study, five of them (*PmLEA8, PmLEA10, PmLEA19, PmLEA20*, and *PmLEA29*) were cloned from cold-resistant *P. mume* ‘Beijingyudie’. Real-time RT-PCR analysis indicated that all these genes could be up-regulated by one or several treatments (ABA, SA, low temperature, high temperature, PEG, and NaCl treatments). The results of spot assay demonstrated that the expression of all these dehydrins, except PmLEA8, conferred improved osmotic and freezing-resistance to the recombinant *Escherichia coli*. So four dehydrin genes, *PmLEA10, PmLEA19, PmLEA20* and *PmLEA29* were chosen for individual over-expression in tobacco plants. The transgenic tobacco plants showed lower relative content of malondialdehyde, relative electrolyte leakage and higher relative content of water than control plants when exposed to cold and drought stress. These results demonstrated that *PmLEAs* were involved in plant responses to cold and drought.

## Introduction

Dehydrins, which are also known as group 2 or D-11 family late-embryogenesis-abundant (LEA) proteins, are a family of highly hydrophilic, glycine-rich, heat-stable, and intrinsically unstructured proteins (Soulages et al., 2003; Hundertmark and Hincha, 2008; Banerjee and Roychoudhury, 2016). They have been reported in a wide range of organisms, including higher plants, mosses, fungi, algae, and cyanobacteria (Close and Lammers, 1993; Cuming et al., 1994; Li et al., 1997; Saavedra et al., 2006; Pochon et al., 2013). Dehydrins typically accumulate in the late stages of seed maturation and in vegetative tissues in response to drought, salinity, low temperature, or abscisic acid (ABA) treatment (Battaglia et al., 2008). Dehydrins are characterized by the presence of one or several conserved, lysine-rich K segment (EKKGIMDKIKEKLPG) (Rorat, 2006). This segment can fold into an amphipathic α-helix structure that may interact with lipid membrane or partially denatured proteins (Koag et al., 2009) to protect the cell against damage caused by stress. Apart from the K segment that can be found in all dehydrins, some dehydrins may possess S segment (serine cluster) and Y segment (DEYGNP). Based on the arrangement of these three segments, dehydrins can be classified into five subclasses: YnSKn, SKn, Kn, YnKn, and KnS (Close, 1996, 1997).

Although the function of dehydrins has not been fully understood, several studies have clearly demonstrated their roles in abiotic stress tolerance. In the study of *Physcomitrella patens*, a knockout dehydrin mutant was generated using homologous recombination. When returned to optimal growth condition after salt and osmotic treatment, the wild type moss could recover to 94% of its fresh weight, while the mutant only reached 39% of its fresh weight (Saavedra et al., 2006). And then, *PpDHNA* and *PpDHNB* whose expressions were induced by salt and osmotic stress were identified in moss. The transgenic *Arabidopsis* plants with overexpressed *PpDHNA* and *PpDHNB* genes showed different effects on rosette and root growth in stress conditions (Ruibal et al., 2012). In cowpea, a 35-kDa dehydrin was demonstrated to cosegregate with chilling tolerance during seedling emergence (Ismail et al., 1999). In *Arabidopsis*, simultaneous overexpression of two dehydrins enhanced cold tolerance. The transgenic plants exhibited reduced ion leakage and improved survival than the control plants (Puhakainen et al., 2004). In banana, SK(3)-type dehydrin gene, *MusaDHN-1* was identified, the transgenic banana plants performed improved drought and salt tolerance (Shekhawat et al., 2011). More and more dehydrin genes have been identified in tomato, *Avicennia officinalis*, wheat, maize, barley, rice, *Rhododendron catawbiense, Saussurea involucrata* and so on. The transgenic plants performed improved tolerance in drought or cold stress (Cheng et al., 2002; Park et al., 2006; Brini et al., 2007; Peng et al., 2008; Munoz-Mayor et al., 2012; Jyothi-Prakash et al., 2014; Kosava et al., 2014; Qiu et al., 2014).

*Prunus mume* Sieb. et Zucc. is a deciduous tree that produces fragrant flowers in late winter to early spring of China. It originated in the southern China around the Yangtze River, and was later introduced to northern China (Jiang and Chen, 2011). Southern China is warm and wet, with lowest temperature around 0°C and average rainfall exceeding 1,000 mm per year. However, for most part of northern China, the annual precipitation is around 500 mm, and its lowest temperature can drop as low as -20°C (Zhai et al., 2005; Song et al., 2011). Low temperature and water deficiency were considered as the key ecological factors that affected the distribution of *P. mume* (Jiang and Chen, 2011). Therefore, many studies have been carried out to enhance the stress tolerance of *P. mume* since 1957 (Chen et al., 2008). The role of dehydrins in *P. mume* stress resistance had never been studied before. Understanding the functions of dehydrins identified from the genomic data of *P. mume*, could provide experimental evidence for future cold/drought-tolerance molecular breeding. In this study, five dehydrin genes were cloned from *P. mume* ‘Beijingyudie’, which has better freezing tolerance than other varieties. Expression and transgenic analyses showed that four of them were involved in stress tolerance.

## Materials and Methods

### Plant Materials and Treatments

*Prunus mume* is also called mei in Chinese. The wild mei found in Tibet was used for genome sequencing (Zhang et al., 2012). *Prunus mume* ‘Beijingyudie’ is a new cultivar that can withstand temperatures as low as -19°C (Chen et al., 1995). Annual branches approximately 60 cm in length with newly mature leaves were collected at 10 am from a 10-year-old ‘Beijingyudie’ tree on the campus of Beijing Forestry University, Beijing, China. Stress treatments were performed following Liu et al. (2009). Approximately 12 branches were placed in flasks containing 200 mM NaCl or 20% PEG-6000 for imposing salt or osmotic stress, respectively. Branches in water served as controls. For the temperature treatments, branches were maintained in distilled water at 4°C or 37°C for low and high temperature treatments, respectively. Branches maintained at 20°C served as controls. For the ABA treatment, branches were transferred to flasks containing 100 μM ABA, and for the SA treatment, branches were treated with 2 mM SA. Untreated branches were used as controls. The leaves were sampled 4 h after treatment. Approximately 3 leaves from different branches were sampled, and immediately frozen in liquid nitrogen and stored in a -80°C freezer until use. For each treatment, 3 independent replications were performed.

### Gene Cloning and Plasmid Construction

Total RNA was extracted from leaves using Trizol reagent (Invitrogen, USA) following the manufacturer’s instructions, and then treated with RNase-free Dnase (Promega). First-strand cDNAs were synthesized from the total RNAs using M-MLV reverse transcriptase (Promega). The coding sequences were cloned using RT-PCR (see **Table 1** for primers). The amplicons were inserted into the GATEWAY donor vector pGWC (Chen et al., 2006), and at least three independent clones were sequenced. The *Escherichia coli* expression vectors (*pDEST15-PmLEAs*) and plant expression vectors (*pEarleyGate203-PmLEAs*) (Earley et al., 2006) were established by LR recombination reaction (Invitrogen, USA) and confirmed by sequencing. All of the bacterial strains mentioned above were only used in the laboratory under controlled conditions.

**Table 1:** Primers sequences used in this study.

Name	Sequence	Utilization
PmLEA8-F	ACCATGGCGAATTACCAGAACC	Gene cloning
PmLEA8-R	TTAATAATGTCGTCCATCAAGTTT	Gene cloning
PmLEA10-F	ACCATGGCGAGCTATGAGAAGC	Gene cloning
PmLEA10-R	CTAGTGTTGTCCGGGAAGTTTC	Gene cloning
PmLEA19-F	ACCATGGCAGATCATTACCCAAAAG	Gene cloning
PmLEA19-R	CTAATACTCCTTTGGCTTCTCC	Gene cloning
PmLEA20-F	ACCATGGCTCAAATTCGTGATGAG	Gene cloning
PmLEA20-R	TTAGTGGCTGTGATGACCAGG	Gene cloning
PmLEA29-F	ACCATGGCGGAGGAGTACAACAA	Gene cloning
PmLEA29-R	TTAATAGGAAGACGTTTCCTTCTC	Gene cloning
PmLEA8-QF	TGCATCTTACGATGGAACCGGCTA	Real-time PCR
PmLEA8-QR	TGGTGGTGGCGGTTGTATGAGTAT	Real-time PCR
PmLEA10-QF	TACAACTGCCACCACCACACCTTA	Real-time PCR
PmLEA10-QR	AGTGTTGTCCGGGAAGTTTCTCCT	Real-time PCR
PmLEA19-QF	AGGAAGGTCAAGGTTGTGGGATGT	Real-time PCR
PmLEA19-QR	CGGCAAGAGTGTGCTTATGTTGCT	Real-time PCR
PmLEA20-QF	ATTGACTGGTGGGAAGCACAAGGA	Real-time PCR
PmLEA20-QR	TCAGTTGTCGCTGTGGTTGTGATG	Real-time PCR
PmLEA29-QF	ATGCTGAGCCTGCAGTAGTAGGTT	Real-time PCR
PmLEA29-QR	TCCTTCTCCTTGATGGCCTCCTTT	Real-time PCR
PmActin2-QF	CCCTAAGGCTAACAGAGAAAAGA	Real-time PCR
PmActin2-QR	CAGCAAGGTCCAGACGAAGAAT	Real-time PCR
NbTubA1-QF	CCTCCTATGCTCCTGTCATTTCAG	Real-time PCR
NbTubA1-QR	ATGGCGAGGATCACACTTAACCA	Real-time PCR
17-F	CAGAAAGAATGCTGACCCAC	Transgenic detection
17-R	GCCATAAAGTCAAAGCCTGC	Transgenic detection

### Gene Expression Analysis

For gene transcript analysis, quantitative real-time RT-PCR was performed using the PikoReal real-time PCR system (Thermo Scientific). The *P. mume* Actin (ID: *Pm005252*) or *Nb Tubulin* (ID: 104112550) gene was used as an internal control (Wang et al., 2014). The specificity of PCR reaction was verified by melting curve analysis. The primers used in the real-time PCR are shown in **Table 1**. Three biological replicates were performed, and each replicate was measured in triplicate. Relative gene expression was calculated according to the ΔΔCT method (Schmittgen and Livak, 2008) and normalized with reference genes and levels under the control condition. For analysis of *PmLEAs* expressing in transgenic tobacco plants, because no specific signal was detected in control (transformed with empty vector), ΔCT method was used to calculate the levels of PmLEAs genes relative to *Nb tubulin*.

### Protein Expression in *E. coli* and Spot Assay

The *pDEST15-PmLEAs* vectors were introduced into *E. coli* strain BL21 (DE3), and the expression of recombinant proteins was induced by 1 mM isopropyl-β-D-thiogalactopyranoside (IPTG) for 1–5 h at 37°C. Protein profiles were examined by 12% SDS-PAGE. After induction by 1mM IPTG for 1 h at 37°C, the spot assay was performed to test the stress tolerance of recombinant *E. coli*, with three replicates for each sample. To evaluate osmotic stress, cell cultures of BL/*pDEST15-PmLEAs* were adjusted to OD600 = 0.6 and then diluted serially (to 1:10, 1:100, and 1:1000). Five microliters of each sample was spotted onto the LB plates containing 1 mM IPTG and 1 M sorbitol. For the freeze-thaw test, 1 ml OD-adjusted cell cultures in tubes were immediately placed in a -80°C freezer and maintained there for 4 h. Then, the samples were thawed at room temperature. After six freeze-thaw cycles, the samples were diluted and spotted onto LB plates with 1 mM IPTG. The plates were incubated at 37°C for 10 h. The bacterial colonies were counted (cfu), and differences were analyzed. BL/*pDEST15-GUS* was used as a control.

### Tobacco Transformation and Stress Tolerance Analysis

The constructed plant expression vectors, *pEarleyGate203-PmLEAs*, were transformed into *Agrobacterium tumefaciens* strain EHA105 using freeze-thaw transformation (Chen et al., 1994). Transgenic tobacco (*Nicotiana benthamiana*) plants were generated by *Agrobacterium*-mediated leaf disk transformation (Burow et al., 1990). Transgenic plants were selected on MS medium supplemented with 2 g/L Basta, and then verified by PCR. Surface-sterilized transgenic seeds (T1) were germinated on 1/2 MS medium containing 2 g/L Basta, and transgenic lines with a 3:1 (resistant: sensitive) segregation ratio were selected to produce seeds. The T2 lines that displayed 100% Basta resistance were selected for further study. Real-time RT-PCR was used to examine the expression of *PmLEAs* in the transgenic lines using α-tubulin gene (*TubA1*, gi: 17402466) as the reference.

The T2 transgenic lines were grown at 22°C under a 16-h light (100 μmol m^-2^s^-1^) and 8-h dark regime with 60% air relative humidity in soil for 2 months. And then the plants were treated in incubator at 4°C for 24 h with 16-h light and 8-h dark to impose cold stress. For the drought treatment, water was withheld from transgenic tobacco plants for 15 days. Before the drought treatment, each pot soil has absorbed water in a tray to saturation. Lines transformed with empty vector were used as controls. Three biological replicates were used to perform one independent experiment and 5 plants for each line were used in one biological replicate. So, approximately 15 plants were included in each line for different treatment. Relative malondialdehyde (MDA) content and relative electrolyte leakage (REL) of leaf samples were tested following the methods of Xing (Xing et al., 2011). The latest two mature leaves from a plant were collected for experiments.

All of the transgenic tobacco plants were grown in a light incubator under controlled conditions.

### Protein Assay and Analysis

The Myc tag was fused with N-terminal of PmLEA protein in plant expression vector *pEarleyGate203-PmLEA.* The target protein accumulations in *PmLEAs* transgenic tobacco plants were detected by western blot using anti-Myc antibody. The leaves were collected from the 2-month-old T2 transgenic tobacco plants and the total proteins were extracted using the grinding buffer (50 mM Tris pH7.5, 150 mM NaCl, 10 mM MgCl_2_, 0.1% NP-40, 1 mM DTT and 1 mM PMSF). The protein concentrations were measured by Nanodrop 2000 using the Bradford method. The loading quantity for each sample was 50 μg. Two 10% SDS-PAGE gels were used, one for western blot, the other for coomassie brilliant blue staining. The membrane stained using Ponceau S after the proteins transferring to PVDF blotting membrane. The PVDF membrane softly shaked in blocking buffer (5% BSA) for 2 h then was washed with TTBS buffer (8.8 g NaCl, 20mL Tris-HCl (1M, pH8.0), 0.5 mL Tween-20 and ddH_2_O to total volume 1L) for three times, 5 min for one time. The blocking buffer added anti-Myc antibody (1:1000) was used in primary antibody incubation. After softly shaking for 2 h, the PVDF membrane was washed with TTBS buffer for five times, 5 min for one time. The block buffer added GAM (1:10000, HRP labeled) was used in secondary antibody incubation. After softly shaking for 2 h, the membrane was washed with TTBS buffer for five times, 5 min for one time. The membrane was coated with chemiluminescence substrate (Thermo Scientific Pierce ECL 32106) and the signals of target proteins were detected by chemical luminescence imaging system.

## Relative Water Content (RWC)

Approximately 3–5 leaves from different transgenic tobacco plants were sampled, and weighed to obtain the fresh weight. The leaves were then immediately hydrated to full turgidity for 3–4 h under normal room light and temperature. After hydration, the leaves were removed, rapidly dried of surface moisture and weighed to obtain the turgid weight. The leaves were then oven dried at 80°C for 24 h and weighed to determine the dry weight. The RWC of each sample was calculated as follows:

RWC (sample) = [(FW-DW)/(TW-DW)],

Where FW is the fresh weight, TW is the turgid weight, and DW is the dry weight.

The values of RWC shown in the results were normalized with those of corresponding lines under control conditions. So RWC was calculated as follows:

RWC = RWC (Treat)/RWC (Control),

Where Treat refers to the cold or drought treatment, and Control refers to the control treatment.

### Statistical Approach for MDA and REL

The MDA content or REL of each sample was tested according to the methods of Xing et al. (2011), with three independent repeats.

The values of MDA and REL shown in the results were normalized with those of corresponding lines under control conditions. So MDA and REL were calculated as follows:

Relative MDA content = MDA content (Treat)/MDA content (Control)

REL = REL (Treat)/REL (Control)

Where Treat refers to the cold or drought treatment and Control refers to the control treatment.

## Results

### Cloning and Characterization of *PmLEAs*

**FIGURE 1:**
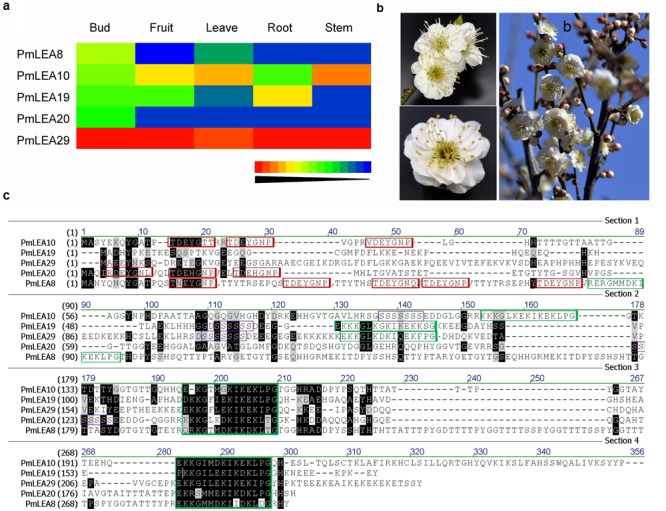
**Identify the PmLEAs from *Prunus mume ‘Beijingyudie’*. (a)** The expressions patterns of *PmLEAs* genes in five organs. It was drawn based transcriptome data. **(b)** The flowers of *Prunus mume ‘Beijingyudie’*. **(c)** The multiple sequences alignment of PmLEAs. The black and gray background show conserved and weakly similar sites respectively. The Y motifs are displayed in the red boxes, the K motifs are in green boxes and the S motifs are in purple boxes.

According to a previous bioinformatics analysis (Du et al., 2013), six dehydrin genes were identified from *P. mume*: *PmLEA8, PmLEA9, PmLEA10, PmLEA19, PmLEA20*, and *PmLEA29*. All of these genes, except *PmLEA9* were cloned from the chosen variety ‘Beijingyudie’ (**Figure 1b**). The expressions patterns of the *PmLEAs* genes in five different organs are shown in **Figure 1a**. The highest expression levels among the genes were observed for *PmLEA29* in all five organs. Although the amino acid sequence homology of the PmLEAs was low (consensus positions: 32.2%; identity positions: 3.1%), they shared some similar segments (**Figure 1c**). The amino acid sequence analysis indicated that *PmLEA8* contains five Y segments and three K segments and therefore belongs to the YnKn-type dehydrins. *PmLEA10* and *PmLEA20* are YnSKn-type dehydrins. *PmLEA19* and *PmLEA29* contained one S segment and three K segments, and therefore belong to SKn-type dehydrins. Compared with the published sequences of *PmLEAs* from the wild mei used for genome sequencing (Zhang et al., 2012), the sequences gained from ‘Beijingyudie’ show some differences at the nucleic acid and amino acid levels (**Table 2**). At the amino acid level, *PmLEA8* in ‘Beijingyudie’ had one variation at position 45 (Q - > H) and two duplications between K segments (**Supplementary Figure S1**). However, for *PmLEA19* and *PmLEA29*, no difference in amino acid sequence between ‘Beijingyudie’ and the wild mei was found.

**Table 2:** Sequence variations of dehydrins in *P. mume* ‘Beijingyudie’.

Gene name	Gene ID^∗^	Nucleotide level	Protein level
PmLEA8	Pm026682	r.135A > C r.292_396dup r.646_699dup	Q45H D98_T132dup P216_T233dup
PmLEA10	Pm026684	r.93G > C r.461A > G r.513G > C	D154G
PmLEA19	Pm020945	No	No
PmLEA20	Pm021811	r.266A > G r.416C > A r.537T > C r.609T > G	N89S T139K
PmLEA29	Pm006114	r.336C > T	No

*^∗^The genome sequences of *Prunus mume* can download from the website: http://prunusmumegenome.bjfu.edu.cn/*

### Transcripts of *PmLEAs* under Different Treatments

**FIGURE 2:**
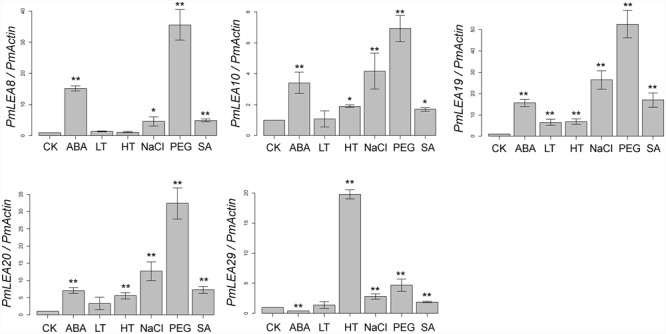
**The relative expression level of *PmLEA*s genes under different treatments.** The *P. mume Actin* was used as a reference gene. Samples in water and maintained at 20°C were used as controls (CK). The data are presented as the mean values of three replicates ± SD. ^∗^, 0.01 < *P* < 0.05; ^∗∗^, *P* < 0.01 (Student’s *t*-test). Three independent experiments were performed with similar results.

The plant hormones ABA and SA play important roles in plant responses to abiotic stresses, such as cold and drought (Cheng et al., 2016; Sah et al., 2016). Real-time RT-PCR was used to examine the transcripts of PmLEAs in response to ABA, SA, low temperature, high temperature, PEG, and NaCl treatments. As shown in **Figure 2**, all of the genes were up-regulated by one or more treatments. However, the expression of *PmLEA29* under the ABA treatment was half that of the control treatment. Generally, the changes of PmLEA10 and PmLEA29 transcriptions after different treatments were lower than those of the other genes. Interestingly, the amount of transcript of *PmLEA29* was increased by more than 19 times under the high temperature treatment relative to the control treatment, whereas under the other treatments, the increases in *PmLEA29* transcription were approximately 4 times the transcript level in the control treatment or lower. Generally, the transcript changes of the dehydrin genes under the ABA, NaCl, and PEG treatments were much greater than the change observed under low temperature. For example, under PEG treatment, all five dehydrin genes were up-regulated more than four times. However, when the branches were exposed to 4°C, only the amount of *PmLEA19* transcript was up-regulated by more than four times. Under SA treatment, the transcripts of *PmLEA8, PmLEA19*, and *PmLEA20* were increased by more than 4 times relative to the control treatment.

### Tolerance of *E. coli* Recombinants Expressing *PmLEAs*

**FIGURE 3:**
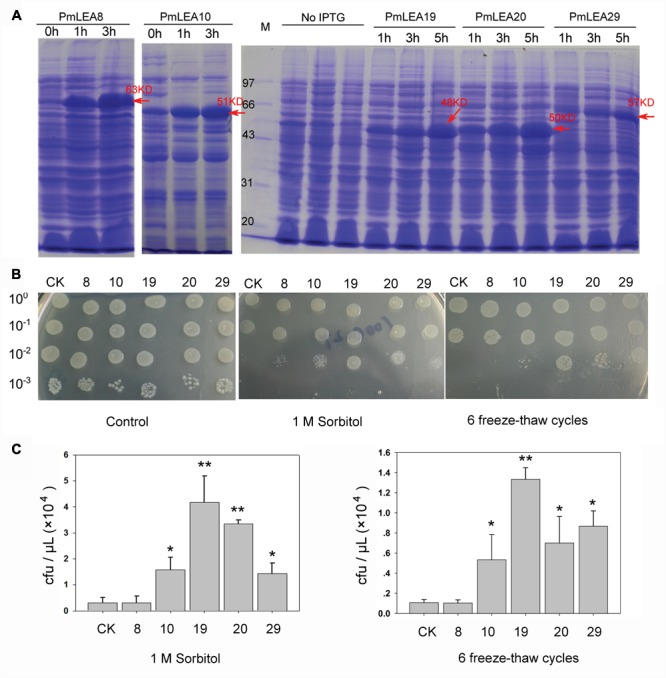
**The prokaryotic expression of proteins PmLEAs and growth performance of *E.coli* under sorbitol or freeze-thaw cycles treatments. (A)** SDS-PAGE analysis of prokaryotic expression product for PmLEAs without IPTG induction (0 h) or subjected to different time of IPTG induction (1 – 5 h). Arrows indicate presence of the induced protein at the expected size. **(B)** The growth performance of E. coli BL21(DE3)/pDEST-PmLEAs on LB plate (left), LB plate with 1M sorbitol (middle) and on LB plate after 6 freeze-thaw cycles (right). CK: BL(DE3)/pDEST15-GUS, 8: BL21(DE3)/pDEST-PmLEA8, 10: BL21(DE3)/pDEST-PmLEA10, 19: BL21(DE3)/pDEST-PmLEA19, 20: BL21(DE3)/pDEST-PmLEA20, 29: BL21(DE3)/pDEST-PmLEA29. The cell cultures were adjusted to OD600 = 0.6, and then diluted serially (1:10, 1:100, and 1:1000, respectively). Five microliter of each sample was spotted onto the LB plates containing 1 mM IPTG. **(C)** The statistical analysis of bacterial colonies after different treatments, as shown in above. The data are presented as the mean values of three replicates ± SD. ^∗^, 0.01 < *P* < 0.05; ^∗∗^, *P* < 0.01 (Student’s *t*-test). Three independent experiments were performed and gave similar results.

*PmLEAs* were introduced into *E. coli* to analyze their functions under different stresses. *E. coli* BL21 harboring *pDEST15-PmLEAs* (BL/*pDEST15-PmLEAs*) were induced with 1 mM IPTG for 1 to 5 h. **Figure 3A** and **Supplementary Figure S5** shows the SDS-PAGE analysis of recombinant *PmLEAs* in *E. coli*. As the pDEST15 vector contains a GST tag, the size of the recombinant proteins of *PmLEA8, PmLEA10, PmLEA19, PmLEA20*, and *PmLEA29* are expected to be approximately 63, 51, 48, 50, and 57 kD, respectively. The size of the bands detected by SDS-PAGE analysis was consistent with the expected size. However, similar-size bands were absent in non-induced cells. The expression levels of the recombinant proteins increased with increasing induction time.

The spot assay was used to determine the effects of *PmLEAs* on the survival of *E. coli* under desiccation and freeze stress. As shown in **Figures 3B,C**, there was no obvious difference in growth behavior between BL/*pDEST15-GUS* and BL/*pDEST15-PmLEAs* on the LB plates, indicating that overexpression of the *PmLEAs* did not inhibit the growth of the *E. coli* recombinants. On LB plates supplemented with 1 M sorbitol, the numbers of BL/*pDEST15-PmLEAs* colonies were much higher than those of BL/*pDEST15-GUS* colonies. However, the protective effect differed among *PmLEAs*. The number of BL/*pDEST15-PmLEA8* colonies was much lower than the numbers of other BL/ *pDEST15-PmLEAs* colonies. Repeated freeze-thaw was used to examine the protective effects of *PmLEAs* on *E. coli* under freeze stress. After six freeze-thaw cycles, all *PmLEA* transformants except *PmLEA8* displayed significantly higher survival than that of control. However, it appeared that the protective effect of *PmLEA10* was weaker than the protective effects of *PmLEA19* and *PmLEA29*.

### Effects of *PmLEA* Overexpression on Cold and Drought Tolerance

**FIGURE 4:**
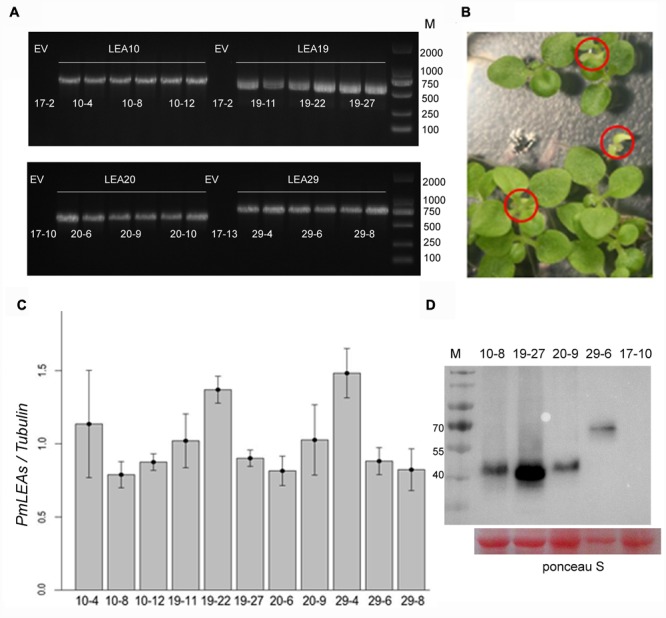
**Identified the expressions of *PmLEAs* in transgenic tobacco plants. (A)** Identified the T1 seedlings of transgenic tobacco lines with PCR. EV: transgenic plant with empty vector. There are six different lines for each gene. **(B)** One of the transgenic T2 lines showing a segregation of Basta resistance. Sensitive plants are displayed in red cycles. **(C)** The expression levels of *PmLEA*s relative to that of α-tubulin in T2 lines. **(D)** The expression of PmLEAs protein in the different transgenic tobacco plants. The proteins were detected using anti-Myc antibody. The ponceau S stain was used as a loading control.

To investigate the functions of *PmLEAs* in stress tolerance, tobacco plants were transformed with *PmLEA10, PmLEA19, PmLEA20*, and *PmLEA29*. *PmLEA8* was excluded from transgenic analysis because of it had the lowest protection effect on *E. coli*. More than fifty independent transgenic tobacco plants (T1) were obtained for each dehydrin gene. Transgenic tobacco plants were confirmed by antibiotic resistance and by amplifying fragments of the *PmLEAs* using PCR (**Figure 4A**). For each gene, the seeds of 12 PCR-confirmed T1 plants were germinated on plates containing Basta. Among them, 29 transgenic T2 lines showed a segregation ratio of 3:1 (resistant: sensitive, **Figure 4B**). To compare the protective effect of different *PmLEA*s, 11 transgenic lines (T2) that showed similar expression levels as α-tubulin were selected for stress tolerance testing: three lines for *PmLEA10* (10-4, 10-8, and 10-12), three lines for *PmLEA19* (19-11, 19-22, and 19-27), two lines for *PmLEA20* (20-6 and 20-9), and three lines for *PmLEA29* (29-4, 29-6, and 29-8) (**Figure 4C**). Three lines transformed with an empty vector (17-2, 17-10, and 17-13), which did not amplify the bands of the recombinant proteins in the gene expression analysis, were used as controls. To confirm the primers used for realtime PCR were specific to target genes and display the homology of dehydrins from tobacco and *P. mume*, the nucleotide and amino acid sequences of dehydrins identified from tobacco and *P. mume*, were compared by multiple sequence alignment (**Supplementary Figures S3 and S4**). The primers located in the specific regions of dehydrins of *P. mume* (**Supplementary Figure S3**). Although the amino acid sequence homology of these dehydrins was low (consensus positions: 28.4%; identity positions: 2.5%), they shared some similar motifs (**Supplementary Figure S4**). To confirm the PmLEAs proteins accumulation in the transgenic tobacco plants, one of each *PmLEAs* transgenic line was selected to detect the level of PmLEA protein respectively using the anti-Myc antibody (**Figure 4D**; **Supplementary Figure S8**). The levels of PmLEAs proteins expressed in transgenic plants were no different, except the PmLEA19 protein with higher level in transgenic line 19–27.

**FIGURE 5:**
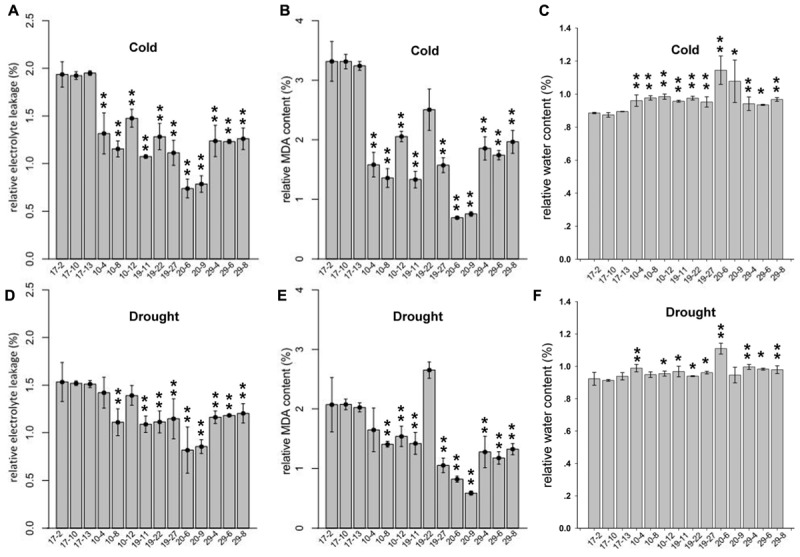
**Relative electrolyte leakage (REL) (A,D)**, relative MDA content **(B,E)** and relative water content **(C,F)** in *PmLEAs* transgenic tobacco lines after cold or drought treatments. Two-month-old transgenic lines were treated at 4°C for 24 h for cold stress. For drought treatment, water was withheld from transgenic tobaccos for 15 days. Three lines for *PmLEA10* (10-4, 10-8, and 10-12), three lines for *PmLEA19* (19-11, 19-22, and 19-27), two lines for *PmLEA20* (20-6 and 20-9), and three lines for *PmLEA29* (29-4, 29-6, and 29-8) were included. Three Lines transformed with empty vector (17-2, 17-10, and 17-13) were used as control. The relative changes were normalized with levels under normal condition (room temperature and well-watered). The data are presented as the mean values of three replicates ± SD. ^∗^, 0.01 < *P* < 0.05; ^∗∗^, *P* < 0.01 (Student’s *t*-test). Three independent experiments were performed and gave similar results.

For the cold-tolerance test, 2-month-old tobacco T2 plants were exposed to 4°C for 24 h. The degree of cold injury was estimated by relative MDA content, REL and RWC. As shown in **Figures 5A–C**, the REL and relative MDA content in transgenic plants were decreased significantly compared with those in the control plants, whereas the RWC was significantly increased in the transgenic tobacco plants. These results suggested that the overexpression of *PmLEAs* enhanced cold tolerance in the transgenic tobacco. The leaves became soft and prolapsed after cold treatment, but there were no obvious differences in phenotypes between the *PmLEAs* transgenic and control plants. For the drought treatment, water was withheld from transgenic tobacco plants for 15 days. The MDA, REL and RWC in the plants were measured. As shown in **Figures 5D–F**, the REL and relative MDA content in *PmLEAs* transgenic plants were decreased compared with those of the control plants, and the RWC was increased in the transgenic plants. It’s interesting that the plants became wilted and the leaves became soft and prolapsed after drought treatment, but there were no obvious differences in phenotypes between the *PmLEAs* transgenic and control plants, although in recovery, all the control plants were dead and the *PmLEAs* transgenic plants showed obviously enhanced drought tolerance compared to the control plants after recoveries by watering (**Supplementary Figure S7**). These results suggested that the overexpression of *PmLEAs* enhanced the resistance to drought stress in transgenic tobacco. Additionally, in the *PmLEA20* transgenic plants, the REL and MDA amounts under normal conditions were significantly higher than those of control plants, whereas the RWC was lower under normal conditions (**Supplementary Figure S2**). When the *PmLEA20* transgenic plants were treated with cold or drought stress, the REL and MDA content were not increased (percentage < 1), and the RWC was not reduced (percentage > 1), compared to the levels in untreated plants (**Figure 5**). It should be mentioned that the size of the *PmLEA20*-overexpressing plants was smaller than that of the control plants (**Supplementary Figure S6**). No morphological or developmental defects were detected for the other transgenic plants.

## Discussion

In this study, we amplified five dehydrin genes (*PmLEA8, PmLEA10, PmLEA19, PmLEA20*, and *PmLEA29*) from *P. mume* ‘Beijingyudie’ based on a previous bioinformatics analysis (Du et al., 2013). For *PmLEA9*, no specific band was found on the agarose gel after several attempts. After inspecting the whole sequence of *PmLEA9*, we found one region that was not completely determined (NNNNN) in intron 2. Therefore, we did not pursue the cloning of *PmLEA9* in this study. Several differences were found between the dehydrin proteins from ‘Beijingyudie’ and those from wild mei in sequencing. For example, *PmLEA8* in ‘Beijingyudie’ has two duplications between K segments at the amino acid level. Similar results have been reported for sunflower (Natali et al., 2003) and wheat (Baloch et al., 2012). Based on the amino acid sequence analysis, the five dehydrin proteins were classified into three groups: YnKn, YnSKn, and SKn. No KnS or Kn type dehydrins were identified in *P. mume*. In apple (*Malus domestica*), 12 dehydrins have been identified and are classified into four groups: YnKn, YnSKn, SKn and Kn (Liang et al., 2012).

The real-time PCR analysis revealed that all of the *PmLEAs* could be induced by different stresses. However, the extent of induction varied among the different treatments, suggesting different roles in *P. mume*. For example, the expression of *PmLEA20* in leaves was very low under normal conditions (Du et al., 2013). However, in the present study, its transcription in leaves was up-regulated by more than four times under most treatments. In contrast to *PmLEA20*, the expression of *PmLEA29* remained at a higher level in different organs under normal conditions (**Figure 1a**), and it was up-regulated by less than four times in most treatments. The phytohormone ABA plays an important role in stress signal transduction (Lee and Luan, 2012). Dehydrins can respond to stresses in either an ABA-independent or ABA-dependent way (Giordani et al., 1999). In this study, *PmLEA8, PmLEA19* and *PmLEA20* were up-regulated by more than four times under ABA treatment, indicating their possible involvement in ABA-dependent pathways. However, *PmLEA29* was not induced by ABA. Similar results have been reported in other studies. For example, in *Arabidopsis, HIRD11* was found to be induced by cold treatment but not by ABA treatment (Hundertmark and Hincha, 2008). In apple, *MdDHN5, MdDHN7*, and *MdDHN8* were not induced by ABA (Liang et al., 2012). Only PmLEA19 was induced by low temperature and can extremely significantly increase the tolerances of recombinant bacteria cells to freeze-thaw treatments and transgenic tobacco plants to cold stress. It means that *PmLEA19* may play an important role in *P*. *mume* to increase the cold tolerance in winter.

Ectopic expression in *E. coli* and tobacco was used to assess the protective effect of *P. mume* dehydrins against stress. Because of the high solubility and few post-translational modifications of dehydrins, the protein structures of dehydrins expressed in *E. coli* were similar to their natural states and may perform the same functions. In our study, the expression of *PmLEA8* protein did not significantly improve the survival of *E. coli* under sorbitol or freeze-thaw treatments. However, the expression of other *PmLEAs* can increase the tolerance of recombinant bacteria cells to sorbitol and freeze-thaw treatments to different degrees. It is suggested that these dehydrins might have undergone functional divergence and yield varying resistance levels under different stresses. Therefore, in this study, the 4 *PmLEAs* (*PmLEA10, PmLEA19, PmLEA20*, and *PmLEA29*) were transformed into tobacco plants to investigate their effects on stress tolerance. The transgenic tobacco plants showed different degrees of tolerances to cold or drought stress in physiological analysis. It is suggested that the dehydrins have similar protective effects in prokaryotic cells and plants. Nevertheless the resistance mechanisms in plants, as eukaryotic multicellular organisms with complex membrane system and metabolic processes, must be more complicated than those in prokaryote cells. In cold treatment, we have extended the time of plants in chilling stress, the leaves of plants were still soft and prolapsed but no obvious differences in phenotypes between the *PmLEAs* transgenic and control plants were found, although significant differences displayed in physiology after cold treatment for 24 h. Lower temperature we will try in our next work to assess the cold tolerance of transgenic plants. In drought treatment, there were no obvious differences in phenotypes between the *PmLEAs* transgenic and control plants, although in recovery, all the control plants were dead and the *PmLEAs* transgenic plants showed obviously enhanced drought tolerance compared to the control plants after recoveries. It’s possible that dehydrins can keep biomembrance and proteins in natural state in plant cells under water deficient, through the hydrophilicity of dehydrins. Although the plants wilted and looked alike in drought stress, the *PmLEAs* transgenic plants could recover growth after watering. The size of *PmLEA20* overexpression plants was smaller than control plants (**Supplementary Figure S6**), and the MDA amounts and electrolyte leakage were significantly higher, the RWC was significantly lower under room temperature than those of control plants (**Supplementary Figure S2**). Considering the low expression of *PmLEA20* in most organs (**Figure 1a**), the overexpression of *PmLEA20* may be harmful to plants. It has been reported in previous studies that the constitutive production of many stress-related proteins can lead to variable levels of growth retardation (Gilmour et al., 2000; Dubouzet et al., 2003; Shekhawat and Ganapathi, 2014). The PmLEA19 protein highly accumulated in transgenic tabacco plants, though it had no significant difference in transcription level compared to other *PmLEAs* genes in transgenic plants. But they had no higher tolerances in *PmLEA19* transgenic lines compared to other *PmLEAs* lines in cold and drought treatments. It means that the tolerance to stress is not simply directly proportional to the accumulation of dehydrin protein; other unkown factors may together affect the tolerances to stresses. It is also possible that the differences in tolerances can not be distinguished by our methods under the existing conditions of experiment and technology.

Dehydrins have the capability to act as hydration buffers due to their hydrophilic segments. They can reduce the water-loss rate of plants in abiotic stress. They can stabilize membranes through the induction of preferential hydration or water replacement at the interface with some macromolecules (Roychoudhury and Nayek, 2014). Lipid membranes are among the most susceptible structures to abiotic stresses. MDA content is widely used as an indicator of lipid peroxidation (Taulavuori et al., 2001). When the tobacco plants were exposed to cold or drought stress, the transgenic lines accumulated less MDA than did the control plants, suggesting that *PmLEAs* can inhibit lipid peroxidation in tobacco. In addition, the REL was lower in transgenic plants than in control plants. As electrolyte leakage occurs following membrane damage, these results indicate that *PmLEAs* can protect membranes from damage. Similar results have been reported in a study of *ZmDHN2b* overexpression in tobacco, wherein *ZmDHN2b*-overexpressing lines had lower levels of cold-induced MDA and less electrolyte leakage than did wild-type tobacco at 4°C (Xing et al., 2011). The contribution of dehydrins to plant abiotic stress tolerance might be partly due to their protective effects on lipid membranes.

## Conclusion

This is the first report of the expression and function of dehydrin genes of *P. mume* under abiotic stresses. Further work is needed to determine how these *PmLEAs* can be used to increase cold and drought tolerance in *P. mume*.

## Author Contributions

FB and DD contributed equally to this work. FB: Conceived and designed the experiments, performed the experiments, analyzed the data, wrote the paper; DD: Conceived and designed the experiments, performed the experiments, analyzed the data, wrote the paper; YA: Performed the experiments; WY: Conceived and designed the experiments; JW: Conceived and designed the experiments; TC: Conceived and designed the experiments; QZ: Conceived and designed the experiments, wrote the paper.

## Conflict of Interest Statement

The authors declare that the research was conducted in the absence of any commercial or financial relationships that could be construed as a potential conflict of interest.

## Supplementary Material

FIGURE S1 **Comparison the amino acid sequences of PmLEA8 from *P. mume* ’Beijingyudie’ and wild type *P. mume*.** Duplicated regions are shown in bold. Y motifs are displayed in the red boxes, and K motifs are underlined.

FIGURE S2 **The absolute MDA contents of *PmLEAs* transgenic tobacco lines in normal conditions.** ^∗^, 0.01 < *P* < 0.05; ^∗∗^, *P* < 0.01 (Student’s *t*-test).

FIGURE S3 **The multiple sequences alignment of dehydrin genes identified from *Nicotiana tabacum* and *Prunus mume*.** The sequences of primers used for realtime PCR are displayed in the red boxes. *NbLEA-Like* (LOC107776344); *NbHIRD11-like* (LOC107797981); *NbHIRD11-like2* (LOC107761179); *NbECPP44-like* (LOC107793843); *NbDHN1-like* (LOC107763252); *NbCOR47-like1* (LOC107820757); *NbCOR47-like2* (LOC107819804).

FIGURE S4 **The multiple sequences alignment of dehydrin proteins from *Nicotiana tabacum* and *Prunus mume*.** The conserved motifs are displayed in the red boxes.

FIGURE S5 **SDS-PAGE analysis of prokaryotic expression product for PmLEA10**.

FIGURE S6 **The phenotypes of *PmLEA20*-overexpressing transgenic plants**.

FIGURE S7 **The phenotypes of *PmLEAs*-overexpressing transgenic plants which were recovering after drought treatment**.

FIGURE S8 **The coomassie brilliant blue staining of the whole gel corresponds to the wester blot.** It was added as loading control for western blot.

## References

[B1] Baloch M. J. Dunwell J. Khan N. U. Khakwani A. A. Dennet M. Jatoi W. A. (2012). Profiling dehydrin gene sequence and physiological parameters in drought tolerant and susceptible spring wheat cultivars. *Pak. J. Bot.* 44 801–806.

[B2] Banerjee A. Roychoudhury A. (2016). Group II late embryogenesis abundant (LEA) proteins: structural and functional aspects in plant abiotic stress. *Plant Growth Regul.* 79 1–17. 10.1007/s10725-015-0113-3

[B3] Battaglia M. Olvera-Carrillo Y. Garciarrubio A. Campos F. Covarrubias A. A. (2008). The enigmatic LEA proteins and other hydrophilins. *Plant Physiol.* 148 6–24. 10.1104/pp.108.120725 18772351 PMC2528095

[B4] Brini F. Hanin M. Lumbreras V. Amara I. Khoudi H. Hassairi A. (2007). Overexpression of wheat dehydrin DHN5 enhances tolerance to salt and osmotic stress in *Arabidopsis thaliana*. *Plant Cell Rep.* 26 2017–2026. 10.1007/s00299-007-0412-x 17641860

[B5] Burow M. Chlan C. Sen P. Lisca A. Murai N. (1990). High-frequency generation of transgenic tobacco plants after modified leaf disk cocultivation with *Agrobacterium tumefaciens*. *Plant Mol. Biol. Rep.* 8 124–139. 10.1007/BF02669766

[B6] Chen H. Nelson R. S. Sherwood J. L. (1994). Enhanced recovery of transformants of *Agrobacterium tumefaciens* after freeze-thaw transformation and drug selection. *Biotechniques* 16 664–668. 8024787

[B7] Chen J. Zhang Q. Liu W. Hu Y. (1995). Studies on breeding for cold hardiness and regional tests of hardy Mei cultivars. *J. Beijing For. Univ.* 17 42–45.

[B8] Chen Q.-J. Zhou H.-M. Chen J. Wang X.-C. (2006). Using a modified TA cloning method to create entry clones. *Anal. Biochem.* 358 120–125. 10.1016/j.ab.2006.08.015 16970900

[B9] Chen R. Zhang Q. Chen J. (2008). Studies on breeding hardy cultivars of mei flower (*prunus mume*) for gardens in Beijing. *Acta Hortic.* 769 305–311.

[B10] Cheng F. Lu J. Gao M. Shi K. Kong Q. Huang Y. (2016). Redox signaling and CBF-responsive pathway are involved in Salicylic acid-improved photosynthesis and growth under chilling stress in Watermelon. *Front. Plant Sci.* 7:1519 10.3389/fpls.2016.01519 PMC5056192 27777580

[B11] Cheng Z. Targolli J. Huang X. Wu R. (2002). Wheat LEA genes, PMA80 and PMA1959, enhance dehydration tolerance of transgenic rice (*Oryza sativa* L.). *Mol. Breed.* 10 71–82.

[B12] Close T. J. (1996). Dehydrins: emergence of a biochemical role of a family of plant dehydration proteins. *Physiol. Plant.* 97 795–803. 10.1111/j.1399-3054.1996.tb00546.x

[B13] Close T. J. (1997). Dehydrins: a commonality in the response of plants to dehydration and low temperature. *Physiol. Plant.* 100 291–296. 10.1034/j.1399-3054.1997.1000210.x

[B14] Close T. J. Lammers P. J. (1993). An osmotic stress protein of cyanobacteria is immunologically related to plant dehydrins. *Plant Physiol.* 101 773–779. 10.1104/pp.101.3.773 8310057 PMC158690

[B15] Cuming A. C. Robertson M. Close T. Chandler P. (1994). Differential regulation by ABA and GA of the expression of a barley dehydrin gene. *J. Exp. Bot.* 45:20.

[B16] Du D. Zhang Q. Cheng T. Pan H. Yang W. Sun L. (2013). Genome-wide identification and analysis of late embryogenesis abundant (LEA) genes in *Prunus mume*. *Mol. Biol. Rep.* 40 1937–1946. 10.1007/s11033-012-2250-3 23086279

[B17] Dubouzet J. G. Sakuma Y. Ito Y. Kasuga M. Dubouzet E. G. Miura S. (2003). OsDREB genes in rice, *Oryza sativa* L., encode transcription activators that function in drought-, high-salt- and cold-responsive gene expression. *Plant J.* 33 751–763. 10.1046/j.1365-313X.2003.01661.x 12609047

[B18] Earley K. W. Haag J. R. Pontes O. Opper K. Juehne T. Song K. M. (2006). Gateway-compatible vectors for plant functional genomics and proteomics. *Plant J.* 45 616–629. 10.1111/j.1365-313X.2005.02617.x 16441352

[B19] Gilmour S. J. Sebolt A. M. Salazar M. P. Everard J. D. Thomashow M. F. (2000). Overexpression of the *Arabidopsis* CBF3 transcriptional activator mimics multiple biochemical changes associated with cold acclimation. *Plant Physiol.* 124 1854–1865. 10.1104/pp.124.4.1854 11115899 PMC59880

[B20] Giordani T. Natali L. D’Ercole A. Pugliesi C. Fambrini M. Vernieri P. (1999). Expression of a dehydrin gene during embryo development and drought stress in ABA-deficient mutants of sunflower (*Helianthus annuus* L.). *Plant Mol. Biol.* 39 739–748. 10.1023/a:1006194720022 10350088

[B21] Hundertmark M. Hincha D. K. (2008). LEA (late embryogenesis abundant) proteins and their encoding genes in *Arabidopsis thaliana*. *BMC Genomics* 9:118 10.1186/1471-2164-9-118 PMC2292704 18318901

[B22] Ismail A. M. Hall A. E. Close T. J. (1999). Allelic variation of a dehydrin gene cosegregates with chilling tolerance during seedling emergence. *Proc. Natl. Acad. Sci. U.S.A.* 96 13566–13570. 10.1073/pnas.96.23.13566 10557361 PMC23988

[B23] Jiang L. Chen J. (2011). A brief introduction to “transferring *Prunus mume* from south to north”–performance and prospect. *Chin. Landsc. Arch.* 1 46–49.

[B24] Jyothi-Prakash P. A. Mohanty B. Wijaya E. Lim T.-M. Lin Q. Loh C.-S. (2014). Identification of salt gland-associated genes and characterization of a dehydrin from the salt secretor mangrove *Avicennia officinalis*. *BMC Plant Biol.* 14:291 10.1186/s12870-014-0291-6 PMC4247641 25404140

[B25] Koag M.-C. Wilkens S. Fenton R. D. Resnik J. Vo E. Close T. J. (2009). The K-segment of maize DHN1 mediates binding to anionic phospholipid vesicles and concomitant structural changes. *Plant Physiol.* 150 1503–1514. 10.1104/pp.109.136697 19439573 PMC2705017

[B26] Kosava K. Vitamvas P. Prasil I. T. (2014). Wheat and barley dehydrins under cold, drought, and salinity — what can LEA-II protein tell us about plant stress response? *Front. Plant Sci.* 5:343 10.3389/fpls.2014.00343 PMC4089117 25071816

[B27] Lee S. C. Luan S. (2012). ABA signal transduction at the crossroad of biotic and abiotic stress responses. *Plant Cell Environ.* 35 53–60. 10.1111/j.1365-3040.2011.02426.x 21923759

[B28] Li R. Brawley S. H. Close T. J. (1997). Dehydrin-like proteins in fucoid algae. *Phycologia* 36 62–62.

[B29] Liang D. Xia H. Wu S. Ma F. (2012). Genome-wide identification and expression profiling of dehydrin gene family in Malus domestica. *Mol. Biol. Rep.* 39 10759–10768. 10.1007/s11033-012-1968-2 23053973

[B30] Liu J. H. Ban Y. Wen X.-P. Nakajima I. Moriguchi T. (2009). Molecular cloning and expression analysis of an arginine decarboxylase gene from peach (*Prunus persica*). *Gene* 429 10–17. 10.1016/j.gene.2008.10.003 18996450

[B31] Munoz-Mayor A. Pineda B. Garcia-Abellan J. O. Anton T. Garcia-Sogo B. Sanchez-Bel P. (2012). Overexpression of dehydrin tas14 gene improves the osmotic stress imposed by drought and salinity in tomato. *J. Plant Physiol.* 169 459–468. 10.1016/j.jplph.2011.11.018 22226709

[B32] Natali L. Giordani T. Cavallini A. (2003). Sequence variability of a dehydrin gene within *Helianthus annuus*. *Theor. Appl. Genet.* 106 811–818. 10.1007/s00122-002-1093-z 12647054

[B33] Park S. Y. Noh K. J. Yoo J. H. Yu J. W. Lee B. W. Kim J. G. (2006). Rapid up regulation of dehydrin3 and dehydrin4 in response to dehydration is a characteristic of drought tolerant genotypes in barley. *J. Plant Biol.* 49 455–462. 10.1007/BF03031126

[B34] Peng Y. Reyes J. L. Wei H. Yang Y. Karlson D. Covarrubias A. A. (2008). RcDhn5, a cold acclimation responsive dehydrin from Rhododendron catawbiense rescue enzyme activity from dehydration effects in vitro and enhances freezing tolerance in RcDhn 5-overexpressing *Arabidopsis* plants. *Physiol. Plant.* 134 583–597. 10.1111/j.1399-3054.2008.01164.x 19000195

[B35] Pochon S. Simoneau P. Pigné S. Balidas S. Bataillé-Simoneau N. Campion C. (2013). Dehydrin-like proteins in the necrotrophic fungus *Alternaria brassicicola* have a role in plant pathogenesis and stress response. *PLoS ONE* 8:e7513 10.1371/journal.pone.0075143 PMC3788798 24098369

[B36] Puhakainen T. Hess M. W. Makela P. Svensson J. Heino P. Palva E. T. (2004). Overexpression of multiple dehydrin genes enhances tolerance to freezing stress in *Arabidopsis*. *Plant Mol. Biol.* 54 743–753. 10.1023/B:PLAN.0000040903.66496.a4 15356392

[B37] Qiu H. Zhang L. Liu C. He L. Wang A. Liu H. L. (2014). Cloning and characterization of a novel dehydrin gene, SiDhn2, from Saussurea involucrate Kar. Et Kir. *Plant Mol. Biol.* 84 707–718. 10.1007/s11103-013-0164-7 24337866

[B38] Rorat T. (2006). Plant dehydrins - tissue location, structure and function. *Cell. Mol. Biol. Lett.* 11 536–556. 10.2478/s11658-006-0044-0 16983453 PMC6275985

[B39] Roychoudhury A. Nayek S. (2014). “Structural aspects and functional regulation of late embryogeniesis abundant (LEA) genes and proteins conferring abiotic stress tolerance in plants,” in *Abiotic Stress: Role in Sustainable Agriculture, Detrimental Effects and Management Strategies* ed. Annabella F. (New York, NY: Nova Publishers) 43–109.

[B40] Ruibal C. Salamo I. P. Carballo V. Castro A. Bentancor M. Borsani O. (2012). Differential contribution of individual dehydrin genes from *Physcomitrella patens* to salt and osmotic stress tolerance. *Plant Sci.* 190 89–102. 10.1016/j.plantsci.2012.03.009 22608523

[B41] Saavedra L. Svensson J. Carballo V. Izmendi D. Welin B. Vidal S. (2006). A dehydrin gene in *Physcomitrella patens* is required for salt and osmotic stress tolerance. *Plant J.* 45 237–249. 10.1111/j.1365-313X.2005.02603.x 16367967

[B42] Sah S. K. Reddy K. R. Li J. (2016). Abscisis acid and abiotic stress tolerance in crop plants. *Front. Plant Sci.* 7:571 10.3389/fpls.2016.00571 PMC4855980 27200044

[B43] Schmittgen T. D. Livak K. J. (2008). Analyzing real-time PCR data by the comparative CT method. *Nat. Protoc.* 3 1101–1108. 10.1038/nprot.2008.73 18546601

[B44] Shekhawat U. K. Srinivas L. Ganapathi T. R. (2011). MusaDHN-1, a novel multiple stress-inducible SK(3)-type dehydrin gene, contributes affirmatively to drought- and salt-stress tolerance in banana. *Planta* 234 915–932. 10.1007/s00425-011-1455-3 21671068

[B45] Shekhawat U. K. S. Ganapathi T. R. (2014). Transgenic banana plants overexpressing MusabZIP53 display severe growth retardation with enhanced sucrose and polyphenol oxidase activity. *Plant Cell Tissue Organ Cult.* 116 387–402. 10.1007/s11240-013-0414-z

[B46] Song Y. L. Zhao Y. X. Wang C. Y. (2011). Changes of accumulated temperature, growing season and precipitation in the North China Plain from 1961 to 2009. *Acta Meteorol. Sin.* 25 534–543. 10.1007/s13351-011-0412-1

[B47] Soulages J. L. Kim K. Arrese E. L. Walters C. Cushman J. C. (2003). Conformation of a group 2 late embryogenesis abundant protein from soybean. Evidence of poly (L-proline)-type II structure. *Plant Physiol.* 131 963–975. 10.1104/pp.015891 12644649 PMC166862

[B48] Taulavuori E. Hellstrom E. K. Taulavuori K. Laine K. (2001). Comparison of two methods used to analyse lipid peroxidation from *Vaccinium myrtillus* (L.) during snow removal, reacclimation and cold acclimation. *J. Exp. Bot.* 52 2375–2380. 10.1093/jexbot/52.365.2375 11709587

[B49] Wang T. Hao R. J. Pan H. T. Cheng T. R. Zhang Q. X. (2014). Selection of suitable reference genes for quantitative real-time polymerase chain reaction in *Prunus mume* during flowering stages and under different abiotic stress conditions. *J. Am. Soc. Hortic. Sci.* 139 113–122.

[B50] Xing X. Liu Y. Kong X. Liu Y. Li D. (2011). Overexpression of a maize dehydrin gene, ZmDHN2b, in tobacco enhances tolerance to low temperature. *Plant Growth Regul.* 65 109–118. 10.1007/s10725-011-9580-3

[B51] Zhai P. M. Zhang X. B. Wan H. Pan X. H. (2005). Trends in total precipitation and frequency of daily precipitation extremes over China. *J. Clim.* 18 1096–1108. 10.1175/jcli-3318.1

[B52] Zhang Q. Chen W. Sun L. Zhao F. Huang B. Yang W. (2012). The genome of *Prunus mume*. *Nat. Commun.* 3:1318 10.1038/ncomms2290 PMC3535359 23271652

