# Supplementary figures and images for: Overexpression of Prunus mume Dehydrin Genes in Tobacco Enhances Tolerance to Cold and Drought
Source: Front Plant Sci. 2017 Feb 7;8:151. doi: 10.3389/fpls.2017.00151 (PMC5293821; doi:10.3389/fpls.2017.00151)

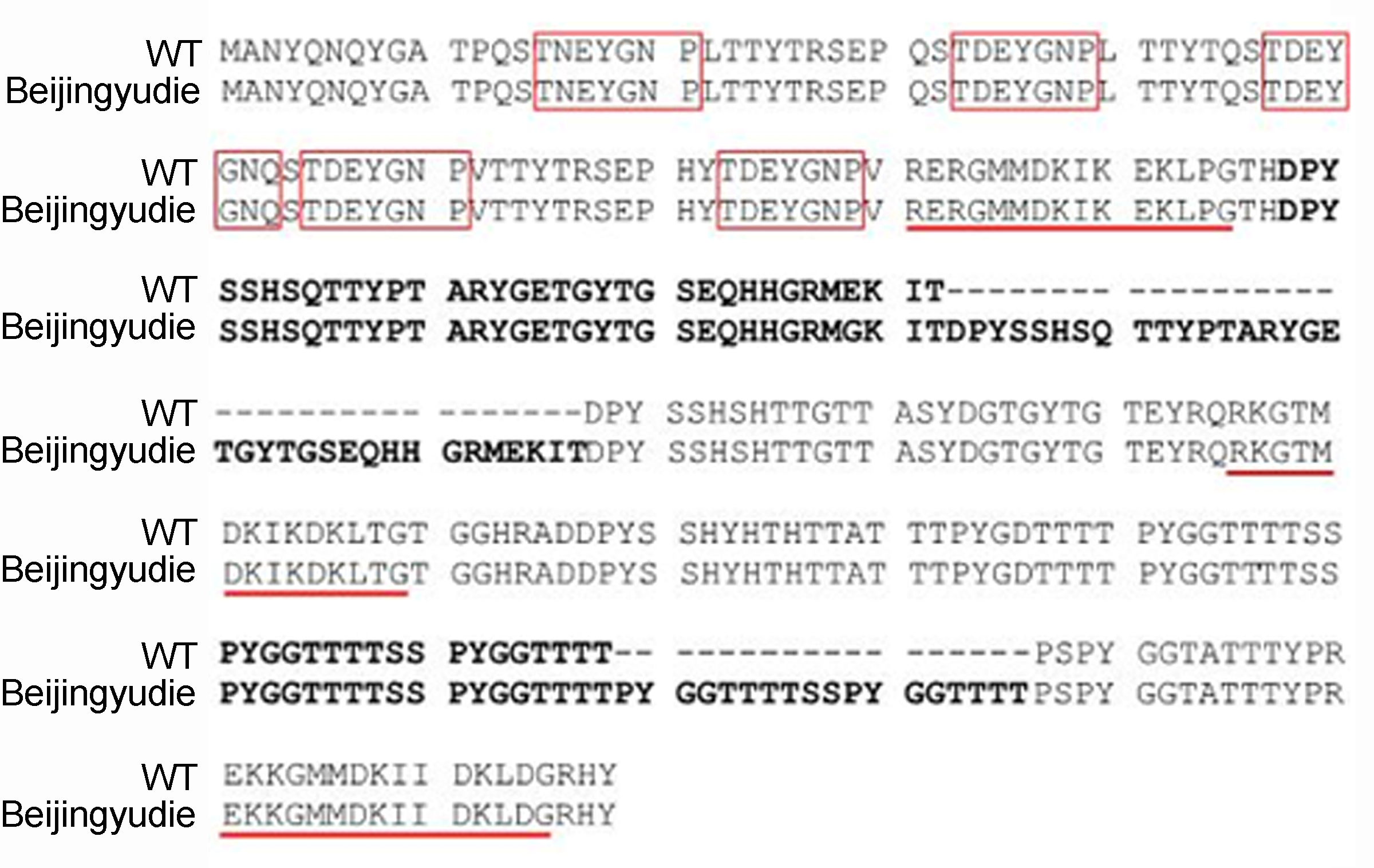

Supplement: FIGURE S1 — Comparison the amino acid sequences of PmLEA8 from P. mume ’Beijingyudie’ and wild type P. mume. Duplicated regions are shown in bold. Y motifs are displayed in the red boxes, and K motifs are underlined. [file Image_1.TIF]

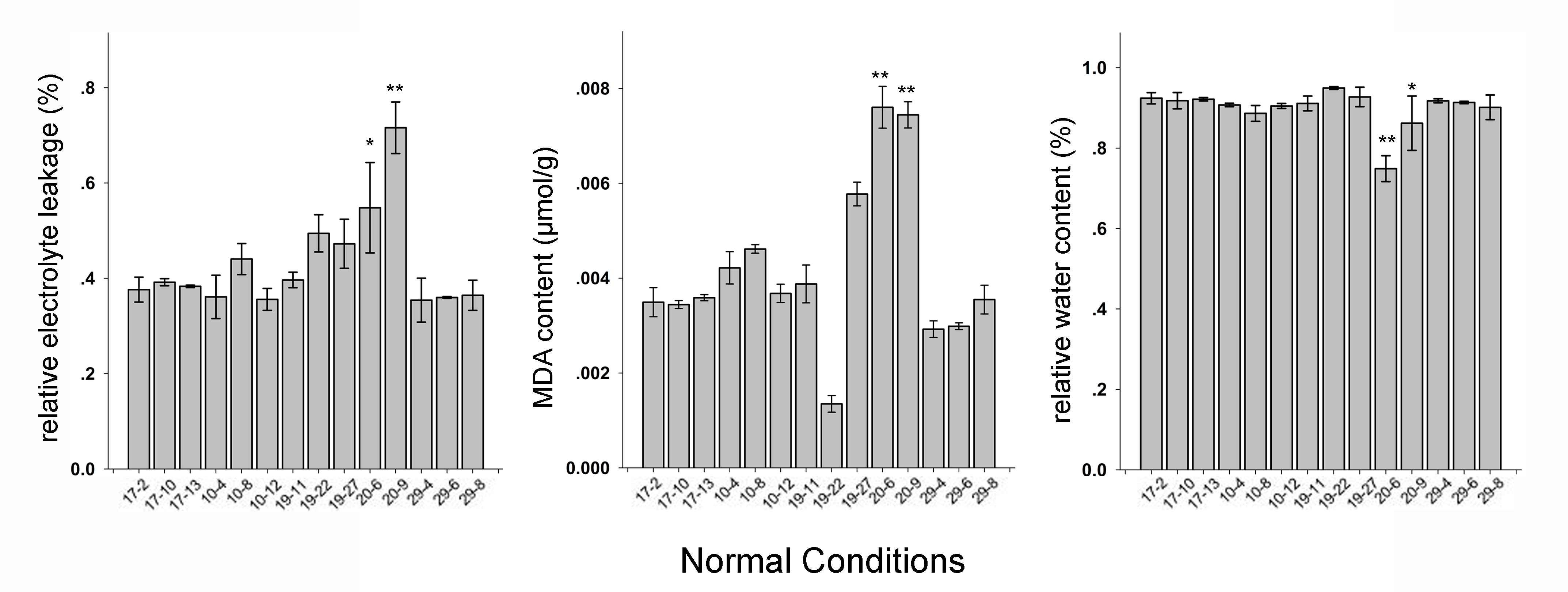

Supplement: FIGURE S2 — The absolute MDA contents of PmLEAs transgenic tobacco lines in normal conditions. ∗, 0.01 < P < 0.05; ∗∗, P < 0.01 (Student’s t-test). [file Image_2.TIF]

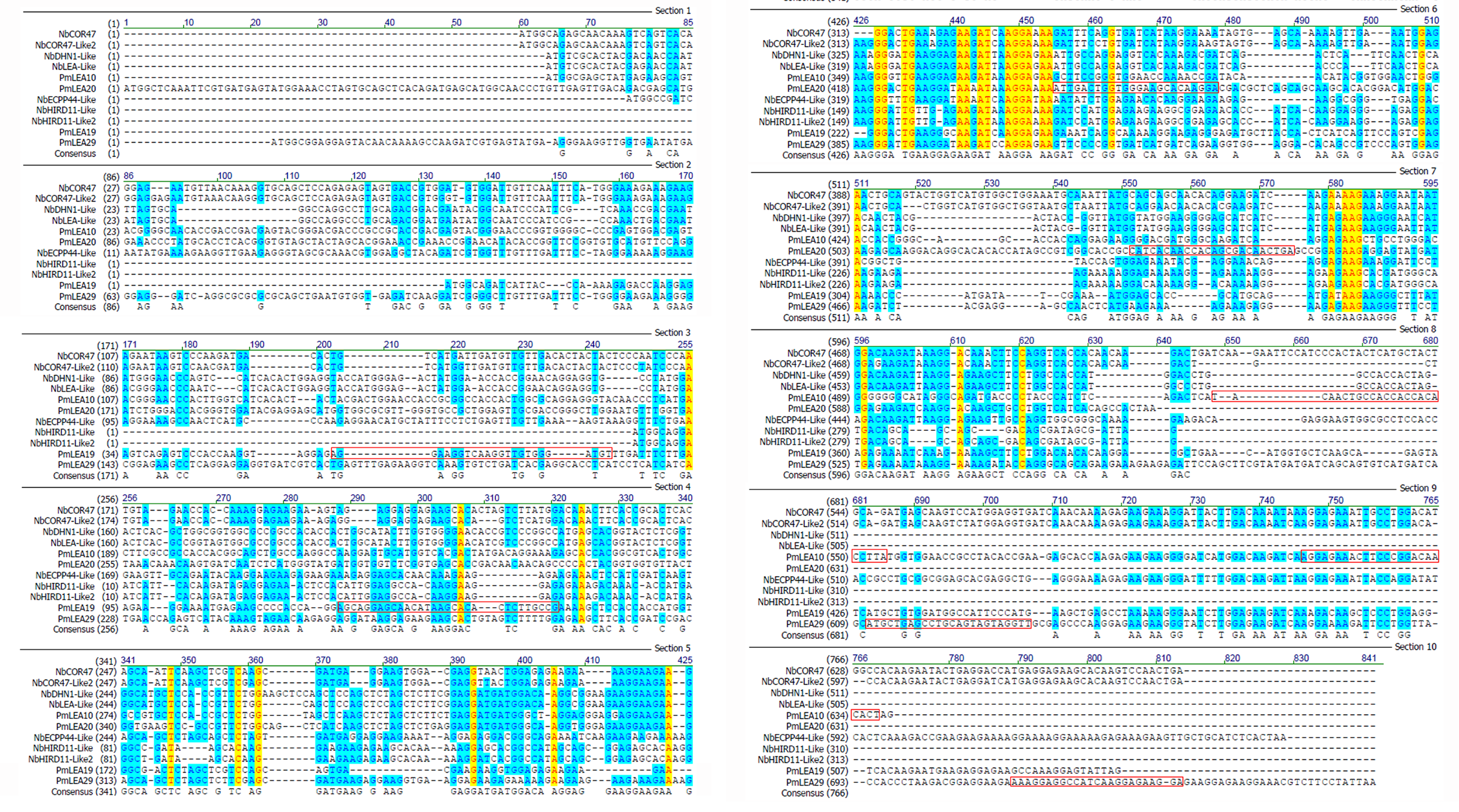

Supplement: FIGURE S3 — The multiple sequences alignment of dehydrin genes identified from Nicotiana tabacum and Prunus mume. The sequences of primers used for realtime PCR are displayed in the red boxes. NbLEA-Like (LOC107776344); NbHIRD11-like (LOC107797981); NbHIRD11-like2 (LOC107761179); NbECPP44-like (LOC107793843); NbDHN1-like (LOC107763252); NbCOR47-like1 (LOC107820757); NbCOR47-like2 (LOC107819804). [file Image_3.TIF]

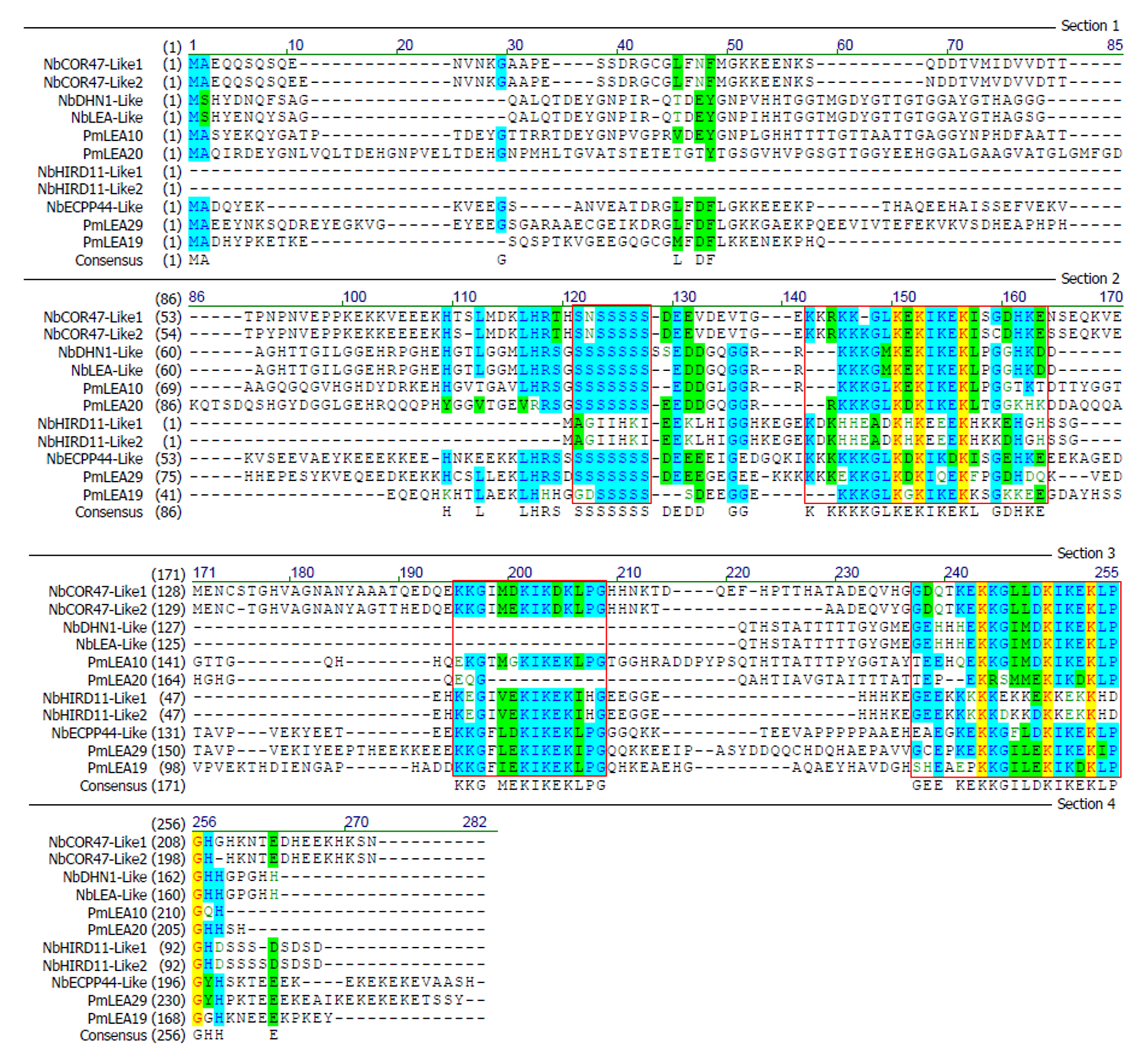

Supplement: FIGURE S4 — The multiple sequences alignment of dehydrin proteins from Nicotiana tabacum and Prunus mume. The conserved motifs are displayed in the red boxes. [file Image_4.TIF]

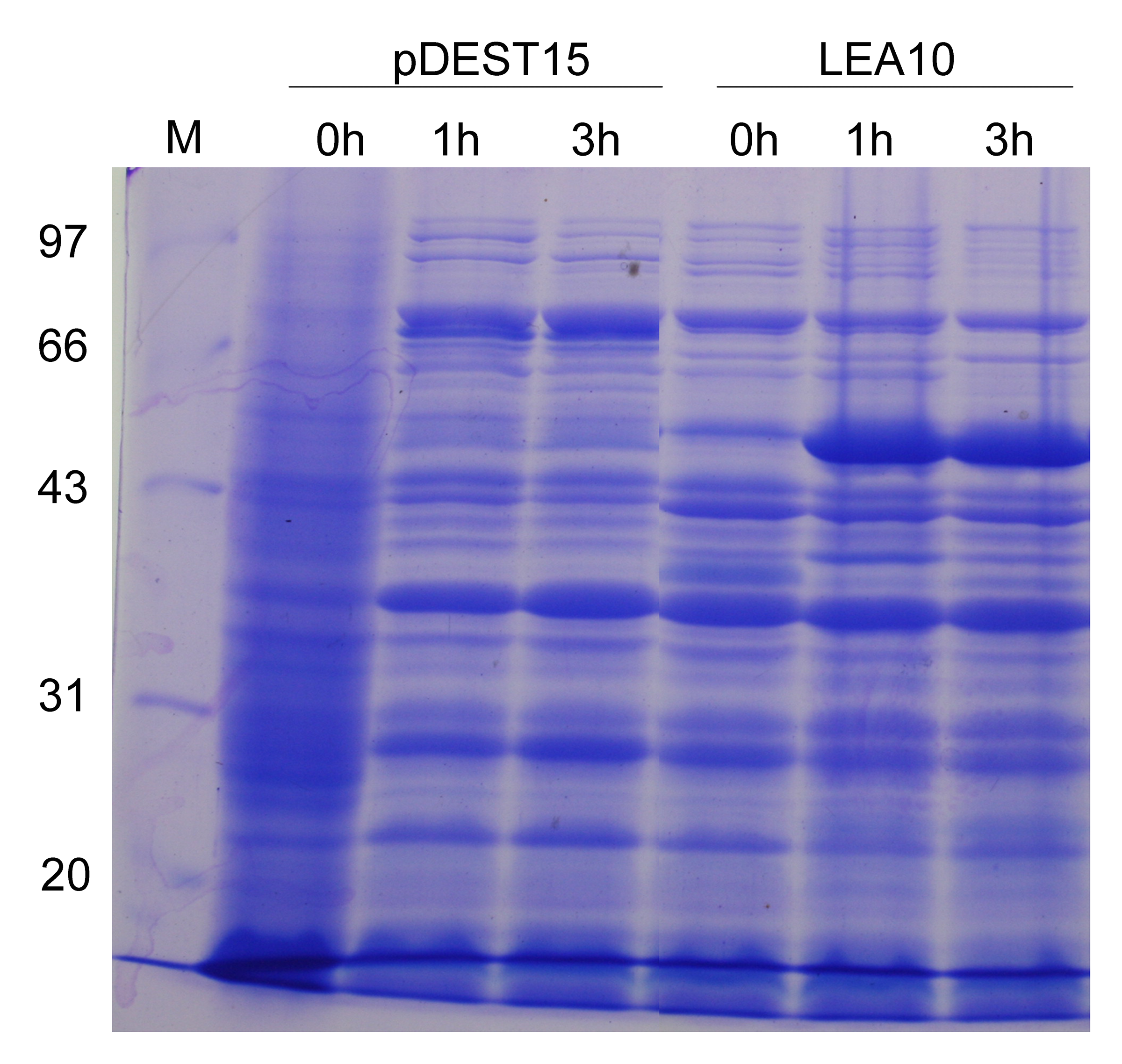

Supplement: FIGURE S5 — SDS-PAGE analysis of prokaryotic expression product for PmLEA10. [file Image_5.TIF]

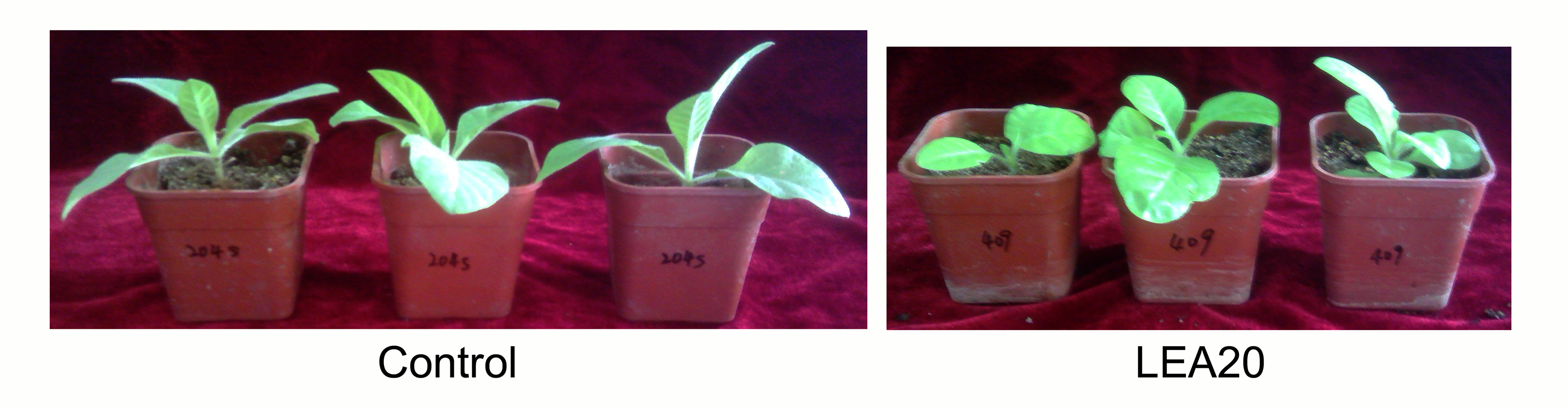

Supplement: FIGURE S6 — The phenotypes of PmLEA20-overexpressing transgenic plants. [file Image_6.TIF]

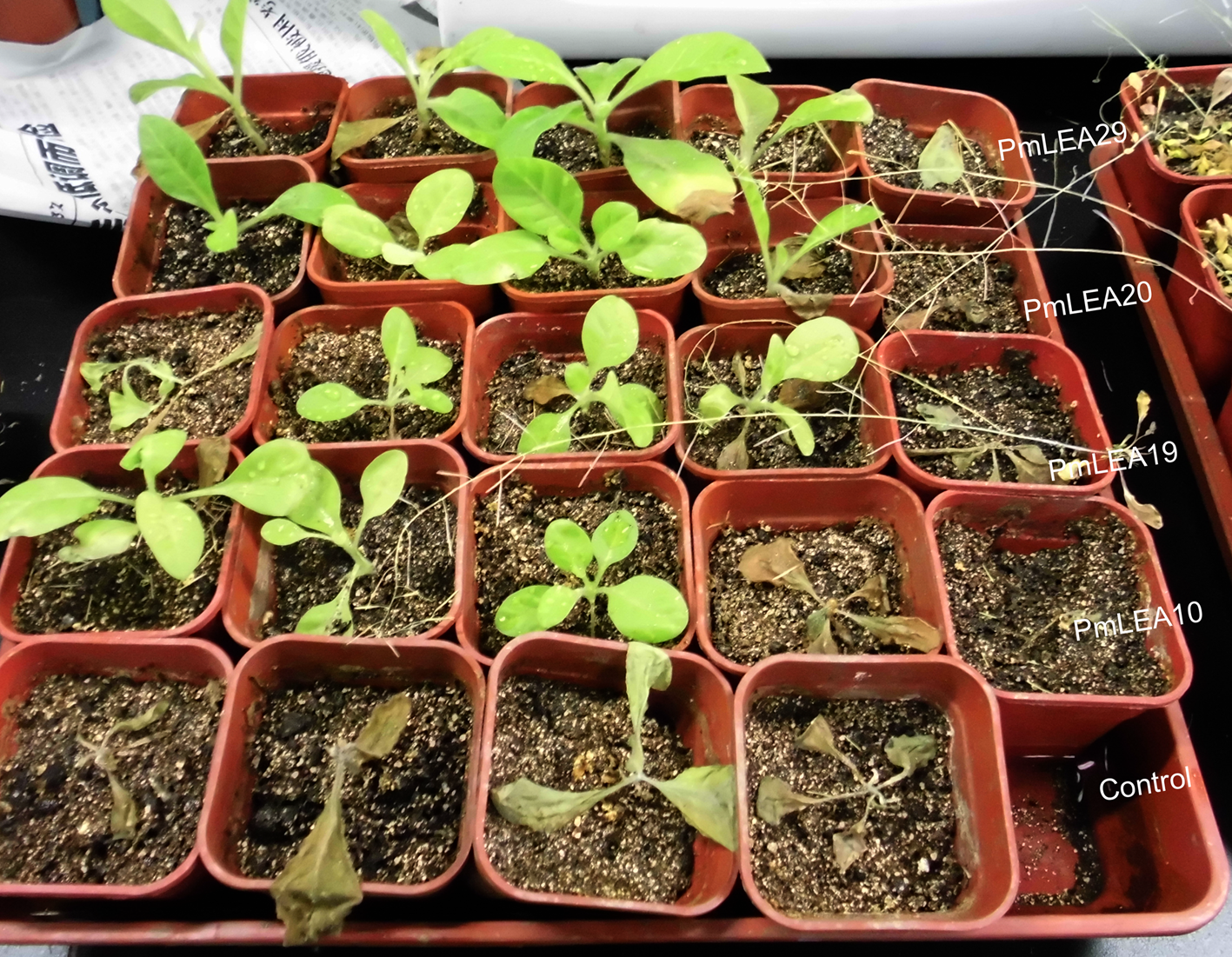

Supplement: FIGURE S7 — The phenotypes of PmLEAs-overexpressing transgenic plants which were recovering after drought treatment. [file Image_7.TIF]

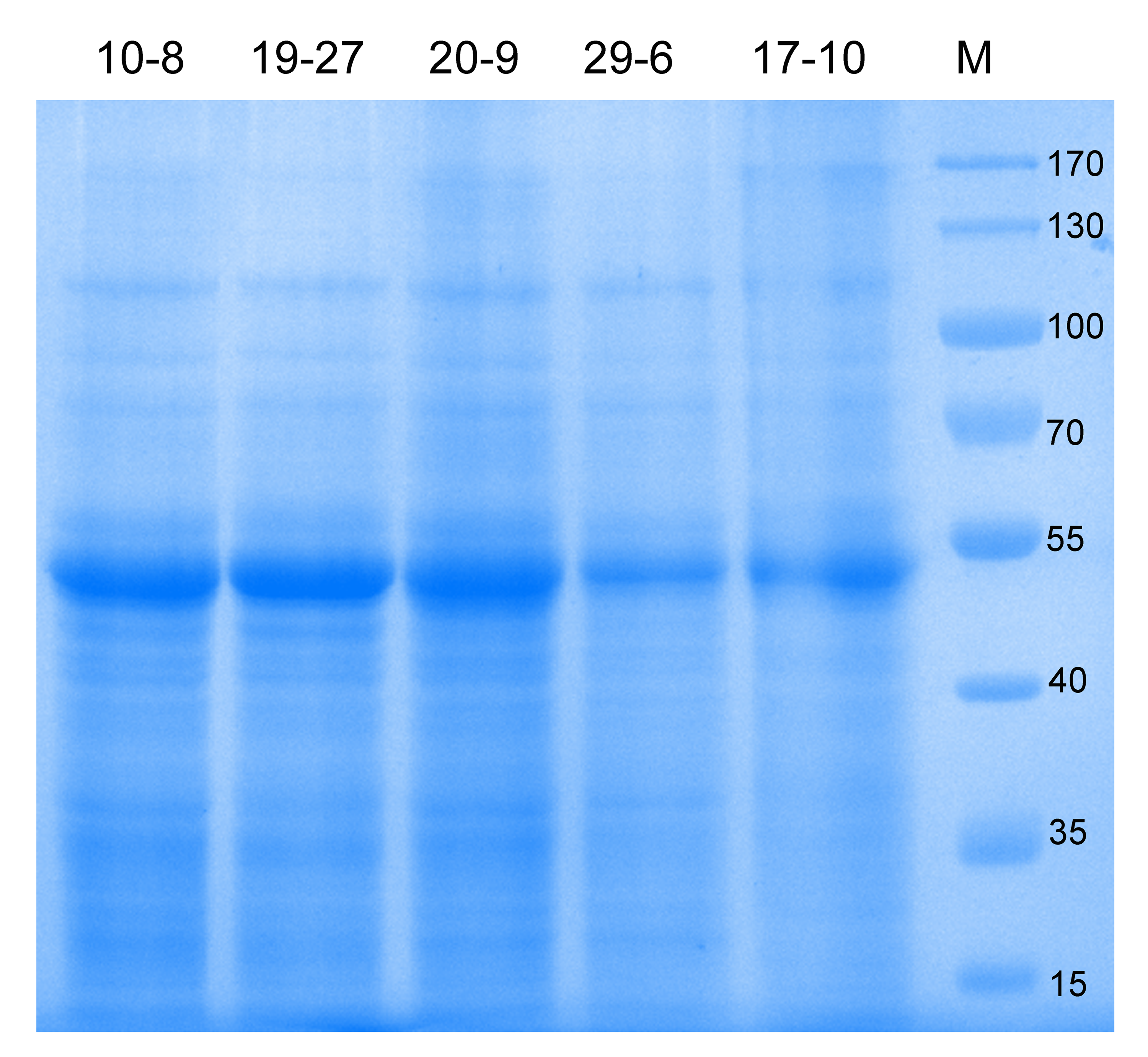

Supplement: FIGURE S8 — The coomassie brilliant blue staining of the whole gel corresponds to the wester blot. It was added as loading control for western blot. [file Image_8.TIF]
